# Supplementary material for: Causal Relationship between Aging and Anorexia Nervosa: A White-Matter-Microstructure-Mediated Mendelian Randomization Analysis
Source: Biomedicines. 2024 Aug 16;12(8):1874. doi: 10.3390/biomedicines12081874 (PMC11351342; doi:10.3390/biomedicines12081874)
Supplement: Supplementary file 1 [file biomedicines-12-01874-s001.zip › Supplementary Figure/Figure S8/Leaveoneout plot of MR analysis for TL to ubm-b-1642.pdf]

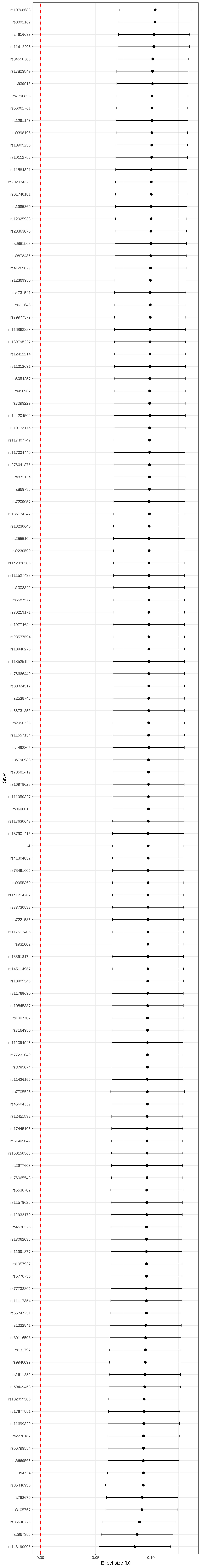
rs10768683
rs3891167
rs4616688
rs11412296
rs34550383
rs17803849
rs939916
rs7790856
rs56061761
rs1291143
rs9398196
rs10905255
rs10112752
rs11584021
rs202034370
rs61748181
rs1985369
rs12925933
rs28363070
rs6881568
rs9878436
rs41269079
rs12369950
rs4731541
rs611646
rs79977579
rs116863223
rs139795227
rs12412214
rs11212631
rs6054257
rs450962
rs7099229
rs144204502
rs10773176
rs117407747
rs117034449
rs376641875
rs871134
rs869785
rs7209057
rs185174247
rs13230646
rs2555104
rs2230590
rs142426306
rs111527438
rs1003322
rs6587577
rs76219171
rs10774624
rs28577594
rs10840270
rs113525195
rs76666449
rs80324517
rs2538745
rs66731853
rs2056726
rs11557154
rs4498805
rs6790988
rs73581419
rs16978028
rs111950327
rs9600019
rs117630647
rs137901416
All
rs41304832
rs78491606
rs9955360
rs141214782
rs73730598
rs7221585
rs117512405
rs932002
rs188918174
rs145114957
rs10805346
rs11769630
rs10845387
rs1907702
rs7164950
rs112394943
rs77231040
rs3785074
rs11426156
rs7705526
rs45604339
rs12451892
rs17445108
rs61405042
rs150150565
rs2977608
rs76065543
rs6536702
rs11579626
rs12932179
rs4530278
rs13062095
rs11991877
rs1957937
rs6776756
rs77732866
rs11117354
rs55747751
rs1332941
rs80116508
rs131797
rs9940099
rs1611236
rs59409453
rs182059586
rs17677991
rs11699829
rs2276182
rs56799554
rs6669563
rs4724
rs35446936
rs762679
rs8105767
rs35640778
rs2967355
rs143190905
SNP
0.00
0.05
0.10
Effect size (b)
